# Supplementary material for: Genome-Based Characterization of Hybrid Shiga Toxin-Producing and Enterotoxigenic Escherichia coli (STEC/ETEC) Strains Isolated in South Korea, 2016–2020
Source: Microorganisms. 2023 May 15;11(5):1285. doi: 10.3390/microorganisms11051285 (PMC10223106; doi:10.3390/microorganisms11051285)
Supplement: Supplementary file 1 [file microorganisms-11-01285-s001.zip › Supplementary Table S5.pdf]

Table S5. Summarized characteristics of pathogenic *E.coli* strains

Strain name	Collection date	Geographic location	Coverage	Contigs	Size (bp)	GC (%)	CDSs	rRNA	tRNA	Accession no.
MFDS1000733	2012-06-28	Incheon	155	317	4,986,545	50.6	5113	8	77	SRR23851467
MFDS1000736	2012-07-10	Incheon	86	293	5,217,997	50.7	5418	4	84	SRR23851453
MFDS1000829	2012-03-30	Daejeon	213	74	4,916,387	50.7	4813	3	77	SRR23851440
MFDS1000830	2012-10-11	Daejeon	217	286	5,103,957	50.5	5292	8	84	SRR23851451
MFDS1001072	2012-06-15	Chungcheongbuk-do	151	330	5,090,733	50.3	5289	7	80	SRR23851434
MFDS1001360	2013-06-24	Gyeongsangbuk-do	250	187	5,049,991	50.3	5182	5	73	SRR23851423
MFDS1001368	2013-06-26	Gwangju	82	271	5,049,628	50.7	5143	6	84	SRR23851412
MFDS1001502	2013-05-30	Incheon	214	206	5,018,758	50.4	5234	9	89	SRR23851411
MFDS1001886	2013-09-30	Busan	55	175	4,994,824	50.5	5055	6	79	SRR23851410
MFDS1002368	2013-12-04	Gangwon-do	93	478	5,071,920	50.5	5319	6	80	SRR23851409
MFDS1002371	2013-12-04	Gangwon-do	86	541	5,242,780	50.7	5412	10	83	SRR23851454
MFDS1002380	2013-12-04	Gangwon-do	80	1166	5,815,734	49.7	6508	10	86	SRR23851455
MFDS1002381	2013-12-04	Gangwon-do	82	1255	5,604,760	49.8	6403	13	84	SRR23851456
MFDS1002417	2013-12-17	Gangwon-do	89	884	5,569,704	50.1	6207	10	78	SRR23851457
MFDS1002425	2013-04-17	Chungcheongnam-do	303	60	4,692,424	50.6	4541	6	75	SRR23851458
MFDS1002429	2013-04-09	Chungcheongnam-do	285	54	4,689,768	50.6	4544	4	71	SRR23851459
MFDS1002430	2013-03-21	Chungcheongnam-do	282	115	4,707,905	50.6	4584	5	78	SRR23851460
MFDS1002932	2013-08-14	Jeollanam-do	282	522	4,955,402	50.5	5168	5	79	SRR23851461
MFDS1002937	2013-07-24	Jeollanam-do	276	154	4,709,289	50.9	4683	4	77	SRR23851462
MFDS1003106	2013-06-28	Incheon	113	385	5,248,488	50.5	5452	6	85	SRR23851463
MFDS1003109	2013-11-05	Incheon	177	176	5,035,365	50.6	4940	6	80	SRR23851441
MFDS1003117	2013-11-12	Incheon	200	329	5,100,598	50.5	5112	6	84	SRR23851464

MFDS1003118	2013-11-27	Gyeonggi-do	205	344	5,090,423	50.5	5105	6	83	SRR23851465
MFDS1003119	2013-11-27	Gyeonggi-do	55	259	5,061,203	50.6	5243	8	82	SRR23851466
MFDS1003120	2013-11-27	Gyeonggi-do	322	324	5,093,703	50.5	5092	12	80	SRR23851445
MFDS1003121	2013-11-26	Incheon	55	837	5,351,232	49.9	5878	11	87	SRR23851446
MFDS1003707	2014-04-22	Incheon	168	197	5,068,613	50.6	5166	4	88	SRR23851447
MFDS1003898	2014-05-28	Incheon	258	228	5,183,006	50.5	5460	4	88	SRR23851448
MFDS1004039	2014-06-16	Jeollanam-do	287	302	5,093,492	50.4	5184	9	84	SRR23851449
MFDS1004047	2014-06-10	Jeollanam-do	269	217	5,014,495	50.4	5034	9	84	SRR23851450
MFDS1004284	2014-02-03	Gwangju	181	297	5,262,232	50.7	5477	8	88	SRR23851452
MFDS1004339	2014-04-14	Gwangju	270	315	5,070,675	50.6	5303	8	83	SRR23851429
MFDS1004787	2014-02-18	Gangwon-do	174	152	5,265,479	50.4	5218	9	86	SRR23851444
MFDS1004796	2014-08-02	Gangwon-do	204	350	5,109,036	50.5	5305	6	87	SRR23851443
MFDS1004800	2014-11-04	Gangwon-do	254	289	5,353,952	50.5	5427	10	94	SRR23851442
MFDS1005423	2015-03-02	Gwangju	167	561	5,518,156	50.3	5993	6	79	SRR23851439
MFDS1005431	2015-03-02	Gwangju	346	1268	5,602,139	50.1	6580	9	91	SRR23851438
MFDS1005534	2015-04-22	Ulsan	189	216	5,255,454	50.6	5334	6	91	SRR23851437
MFDS1005656	2015-04-27	Gwangju	172	370	5,387,389	50.4	5695	8	94	SRR23851436
MFDS1005678	2015-05-25	Gwangju	175	314	5,212,397	50.5	5476	8	85	SRR23851435
MFDS1005756	2015-08-25	Gyeonggi-do	160	279	4,889,956	50.7	4998	8	81	SRR23851433
MFDS1005763	2015-08-28	Gyeonggi-do	152	170	5,038,664	50.6	4948	10	81	SRR23851432
MFDS1006180	2015-10-26	Incheon	113	490	4,863,935	50.6	5158	6	79	SRR23851431
MFDS1006199	2015-05-04	Jeollanam-do	218	343	5,105,114	50.7	5216	9	82	SRR23851430
MFDS1006212	2015-05-23	Jeollanam-do	241	453	5,009,353	50.6	5206	5	79	SRR23851408
MFDS1006458	2015-08-19	Chungcheongnam-do	97	372	4,930,872	50.5	5137	12	65	SRR23851428
MFDS1006494	2015-11-26	Chungcheongnam-do	177	373	5,464,751	50.7	5774	6	83	SRR23851427

MFDS1006657	2015-08-06	Gwangju	209	196	5,099,659	50.7	5177	4	90	SRR23851426
MFDS1006808	2015-06-09	Jeollabuk-do	207	1564	5,791,139	50.6	6966	9	96	SRR23851425
MFDS1006810	2015-06-16	Jeollabuk-do	204	239	5,053,696	50.6	5183	9	87	SRR23851424
MFDS1007039	2016-08-22	Daegu	133	238	4,903,481	50.7	4945	7	83	SRR23851422
MFDS1007042	2016-09-02	Chungcheongbuk-do	225	156	4,779,554	50.6	4729	10	76	SRR23851421
MFDS1007291	2016-05-17	Jeollabuk-do	214	462	5,141,778	50.4	5426	4	61	SRR23851420
MFDS1007294	2016-05-24	Jeollabuk-do	225	911	5,340,735	50.5	5918	5	86	SRR23851419
MFDS1007302	2016-07-04	Gyeonggi-do	226	255	5,199,726	50.6	5406	8	85	SRR23851418
MFDS1007304	2016-07-04	Gyeonggi-do	194	147	4,972,292	50.6	4992	9	84	SRR23851417
MFDS1007314	2016-05-30	Gyeongsangbuk-do	235	640	5,339,017	50.5	5699	8	82	SRR23851416
MFDS1007322	2016-07-18	Gwangju	177	314	4,969,455	50.7	5081	10	83	SRR23851415
MFDS1007440	2016-07-02	Incheon	151	475	5,027,796	50.5	5241	6	82	SRR23851414
MFDS1007774	2016-05-09	Jeollanam-do	265	1212	5,114,715	50.6	5565	4	74	SRR23851413
MFDS1007787	2016-06-15	Jeollanam-do	226	435	4,689,229	50.8	4735	6	72	SRR23867020
MFDS1007792	2016-06-28	Jeollanam-do	232	737	5,413,787	50.6	5864	5	79	SRR23867019
MFDS1007799	2016-07-25	Jeollanam-do	292	598	4,988,758	51	5286	3	76	SRR23867008
MFDS1007807	2016-08-23	Jeollanam-do	225	1444	5,263,382	50.8	5924	4	68	SRR23866997
MFDS1007812	2016-08-30	Jeollanam-do	220	1162	5,309,919	50.8	6003	5	75	SRR23866986
MFDS1007818	2016-09-26	Jeollanam-do	212	464	5,071,985	50.6	5130	4	78	SRR23866975
MFDS1008070	2016-11-22	Daejeon	250	891	5,049,444	50.8	5460	5	74	SRR23866964
MFDS1008074	2016-11-30	Daejeon	214	2793	5,945,913	49.5	7771	9	118	SRR23866963
MFDS1008112	2016-08-22	Seoul	135	146	5,032,981	50.6	5061	6	80	SRR23866962
MFDS1008113	2016-08-22	Seoul	53	1306	6,476,170	50.6	7473	4	89	SRR23866961
MFDS1008157	2016-03-21	Ulsan	234	1105	5,002,886	50.8	5427	6	66	SRR23867018
MFDS1008159	2016-03-03	Ulsan	189	2039	4,500,504	51.2	5834	6	57	SRR23867017

MFDS1008398	2016-04-29	Chungcheongnam-do	193	438	5,093,047	50.5	5309	10	84	SRR23867016
MFDS1008400	2016-06-20	Chungcheongnam-do	229	1047	5,689,783	50.3	6327	9	87	SRR23867015
MFDS1008401	2016-08-22	Chungcheongnam-do	169	382	5,169,262	50.5	5322	8	82	SRR23867014
MFDS1008402	2016-08-22	Chungcheongnam-do	209	473	5,198,164	50.5	5522	5	78	SRR23867013
MFDS1008408	2016-11-22	Chungcheongnam-do	227	304	5,160,252	50.6	5316	8	81	SRR23867012
MFDS1008507	2017-06-15	Jeollanam-do	108	474	5,108,471	50.6	5385	7	81	SRR23867011
MFDS1008634	2017-05-22	Jeollabuk-do	113	423	5,551,194	50.3	5990	7	82	SRR23867010
MFDS1008698	2017-04-13	Gyeongsangbuk-do	303	269	4,948,421	50.6	5035	6	82	SRR23867009
MFDS1009417	2017-09-20	Gyeongsangbuk-do	253	247	4,992,953	50.6	5130	5	78	SRR23867007
MFDS1009477	2016-10-13	Incheon	243	222	5,194,533	50.4	5315	7	78	SRR23867006
MFDS1009478	2016-10-18	Incheon	185	161	4,956,666	50.7	4839	7	82	SRR23867005
MFDS1009481	2016-10-18	Incheon	270	446	5,152,489	50.3	5377	8	76	SRR23867004
MFDS1009482	2016-11-14	Incheon	187	385	5,159,579	50.5	5410	7	81	SRR23867003
MFDS1009483	2017-04-25	Incheon	244	707	4,986,125	50.6	5292	6	70	SRR23867002
MFDS1009493	2017-06-26	Incheon	180	244	4,922,029	50.6	4982	4	81	SRR23867001
MFDS1009513	2017-08-28	Incheon	171	240	5,105,434	50.5	5212	6	84	SRR23867000
MFDS1009528	2017-07-17	Chungcheongbuk-do	179	234	5,309,329	50.4	5545	5	83	SRR23866999
MFDS1009657	2017-07-17	Jeollabuk-do	184	123	5,026,485	50.8	5017	6	85	SRR23866996
MFDS1009673	2017-09-12	Jeollabuk-do	185	280	5,259,307	50.4	5451	9	84	SRR23866995
MFDS1009675	2017-09-18	Jeollabuk-do	270	294	5,070,912	50.5	5233	4	80	SRR23866994
MFDS1009680	2017-10-11	Jeollabuk-do	230	142	5,217,763	50.5	5325	4	79	SRR23866993
MFDS1009685	2017-10-24	Jeollabuk-do	229	404	5,260,771	50.5	5501	8	81	SRR23866992
MFDS1009721	2016-09-20	Incheon	207	1500	4,818,016	50.7	5440	5	67	SRR23866991
MFDS1009723	2016-09-20	Incheon	236	250	5,183,956	50.7	5397	4	79	SRR23866990
MFDS1009729	2017-06-29	Incheon	170	265	5,132,845	50.4	5307	7	77	SRR23866989

MFDS1009732	2017-08-31	Incheon	231	291	5,079,029	50.7	5242	4	87	SRR23866988
MFDS1009733	2017-08-31	Incheon	241	177	5,450,231	50.2	5559	4	81	SRR23866987
MFDS1009735	2017-08-31	Incheon	206	103	4,783,229	50.6	4715	4	71	SRR23866985
MFDS1009740	2017-08-31	Incheon	239	92	4,859,418	50.7	4812	5	73	SRR23866984
MFDS1009742	2017-08-31	Incheon	253	205	5,093,717	50.6	5283	4	75	SRR23866983
MFDS1009747	2017-08-31	Incheon	229	1056	4,926,639	50.6	5281	4	58	SRR23866982
MFDS1009748	2017-08-31	Incheon	221	486	4,913,650	50.7	5055	4	76	SRR23866981
MFDS1009749	2017-08-31	Incheon	258	133	4,925,276	50.7	4952	4	77	SRR23866980
MFDS1009750	2017-08-31	Incheon	201	1622	5,081,024	50.8	5919	3	67	SRR23866979
MFDS1009757	2017-09-08	Incheon	178	187	5,047,310	50.5	5121	7	79	SRR23866978
MFDS1009762	2017-05-20	Incheon	221	528	4,924,469	50.7	5050	5	71	SRR23866977
MFDS1009763	2017-05-20	Incheon	258	362	5,224,631	50.6	5544	6	80	SRR23866976
MFDS1010174	2017-08-18	Gyeongsangnam-do	237	2033	6,114,130	50.3	7819	5	88	SRR23866973
MFDS1010260	2017-10-27	Gyeongsangbuk-do	225	2866	6,204,399	50.6	8354	5	83	SRR23866972
MFDS1010313	2017-08-31	Chungcheongnam-do	216	599	4,980,461	50.6	5169	5	75	SRR23866971
MFDS1010484	2017-05-23	Gwangju	180	513	5,339,897	50.4	5780	7	83	SRR23866970
MFDS1010545	2017-08-28	Jeollabuk-do	181	290	5,388,409	50.3	5642	9	85	SRR23866969
MFDS1010733	2017-12-04	Jeollabuk-do	231	313	5,192,798	50.6	5374	5	83	SRR23866968
MFDS1010859	2017-12-12	Incheon	174	1568	5,041,678	50.6	5938	7	68	SRR23866967
MFDS1010997	2018-02-06	Jeollabuk-do	241	1563	5,060,570	50.7	5911	4	65	SRR23866965
MFDS1011014	2018-03-12	Jeollabuk-do	348	335	5,404,611	50.5	5707	4	83	SRR23867158
MFDS1011026	2018-04-23	Jeollabuk-do	278	339	5,512,231	50.3	5775	7	80	SRR23867157
MFDS1011033	2018-05-08	Jeollabuk-do	211	252	5,318,095	50.6	5560	5	79	SRR23867146
MFDS1011123	2018-05-18	Jeollanam-do	319	376	5,375,775	50.4	5690	3	80	SRR23867135
MFDS1011126	2018-05-18	Incheon	272	239	4,983,492	50.5	5098	6	69	SRR23867124

MFDS1011127	2018-06-19	Incheon	257	232	4,964,708	50.7	5098	6	80	SRR23867120
MFDS1011176	2018-06-04	Jeollabuk-do	269	206	5,314,655	50.7	5406	6	77	SRR23867119
MFDS1011181	2018-06-18	Jeollabuk-do	217	1836	6,373,314	47.4	7818	10	106	SRR23867118
MFDS1011183	2018-06-18	Jeollabuk-do	248	295	5,232,979	50.4	5545	5	83	SRR23867117
MFDS1011624	2018-04-30	Chungcheongbuk-do	283	883	5,883,953	50.4	6578	6	91	SRR23867116
MFDS1012271	2018-07-13	Gyeongsangbuk-do	278	105	4,786,924	50.7	4697	3	68	SRR23867156
MFDS1012272	2018-09-10	Ulsan	194	490	5,202,824	50.4	5552	3	80	SRR23867155
MFDS1012281	2018-08-29	Seoul	249	337	5,343,417	50.7	5447	4	76	SRR23867154
MFDS1012295	2018-10-24	Gyeongsangbuk-do	260	340	5,476,050	50.3	5820	5	92	SRR23867153
MFDS1012304	2018-07-24	Jeollabuk-do	251	258	5,335,178	50.4	5555	3	79	SRR23867152
MFDS1012310	2018-08-21	Jeollabuk-do	237	257	5,365,029	50.8	5562	4	77	SRR23867151
MFDS1012319	2018-09-10	Jeollabuk-do	255	665	5,411,102	50.4	5958	4	81	SRR23867150
MFDS1012327	2018-10-01	Jeollabuk-do	250	191	4,987,158	50.8	5003	4	71	SRR23867149
MFDS1012536	2018-07-23	Chungcheongnam-do	266	892	5,366,853	50.6	5887	8	72	SRR23867148
MFDS1012541	2018-10-26	Chungcheongbuk-do	233	325	5,453,131	50.3	5797	6	89	SRR23867147
MFDS1013275	2019-05-21	Gyeongsangbuk-do	78	165	4,837,872	50.7	4869	7	81	SRR23867145
MFDS1013297	2019-06-21	Daegu	128	970	5,348,510	49.6	6024	11	88	SRR23867144
MFDS1013298	2019-01-31	Seoul	129	1325	5,875,999	48.9	6993	8	91	SRR23867143
MFDS1014133	2019-03-11	Gwangju	103	1413	5,942,014	50.4	7011	7	84	SRR23867142
MFDS1014148	2019-09-19	Gwangju	129	1438	5,692,085	49.1	6776	10	88	SRR23867141
MFDS1014205	2019-06-23	Daejeon	154	342	5,098,967	50.5	5294	11	89	SRR23867140
MFDS1014218	2019-12-31	Gyeongsangnam-do	145	274	5,370,709	50.5	5502	9	84	SRR23867139
MFDS1014288	2019-04-08	Chungcheongbuk-do	200	269	5,364,401	50.4	5639	7	90	SRR23867138
MFDS1016415	2020-07-18	Ulsan	157	312	5,180,935	50.5	5423	10	89	SRR23867137
MFDS1016423	2020-07-27	Gyeongsangbuk-do	106	721	5,029,473	50.1	5430	8	84	SRR23867136

MFDS1016424	2020-07-30	Gyeongsangbuk-do	101	737	5,041,249	50	5474	9	78	SRR23867134
MFDS1018271	2020-06-26	Chungcheongnam-do	117	353	5,019,718	50.5	5215	7	87	SRR23867133
MFDS1019472	2021-07-27	Jeollabuk-do	121	269	4,960,555	50.6	5073	7	86	SRR23867131
MFDS2001049	2016-06-17	Jeju-do	116	340	5,477,558	50.7	5762	5	84	SRR23867130
MFDS2001053	2016-06-17	Jeju-do	81	281	5,388,694	50.6	5617	7	82	SRR23867129
MFDS2001387	2017-05-18	Chungcheongbuk-do	165	210	5,001,955	50.6	5075	8	79	SRR23867128
MFDS2002396	2015-02-01	Busan		5	5,100,852	50.6	5139	22	86	GCA_001750845.1
MFDS2007261	2020-04-13	Missing	328	413	4,922,104	50.7	5206	5	75	SRR23867127
MFDS9000093	2021-08-10	Missing	171	595	4,991,175	50.7	5553	5	85	SRR23867126
MFDS9000105	2021-08-10	Busan	178	617	5,032,528	50.7	5554	5	82	SRR23867125
MFDS9000108	2021-08-10	Missing	152	416	5,062,915	50.5	5320	8	89	SRR23867123
MFDS9000239	2021-08-10	Missing	140	536	4,680,835	50.7	5083	5	78	SRR23867122
MFDS9000240	2021-08-10	Missing	151	883	5,253,593	50.8	5820	7	91	SRR23867121
2009EL-2050	Missing	Missing	-	1	5,253,138	50.7	5220	22	94	GCA_000299255.1
2009EL-2071	Missing	Missing	-	1	5,312,586	50.7	5297	22	98	GCA_000299475.1
2011EL-3493	Missing	Missing	-	1	5,273,097	50.7	5251	22	94	GCA_000299455.1
55989	Missing	Missing	-	2	5,227,344	50.6	5201	22	94	GCA_000026245.1
8_3_Ti3	2010	Australia	-	3	5,141,085	50.8	5337	22	97	GCA_012221565.1
53638	Missing	Missing	-	4	5,371,790	51	5549	22	102	GCA_000167915.2
152661	2014-07	United Kingdom	-	3	5,199,422	50.6	7391	22	83	GCA_009762515.1
CFSAN029787	2012-04-15	Italy	-	3	5,288,947	50.5	5381	22	104	GCA_001007915.1
B171	Missing	Missing	-	265	5,654,428	50.5	6124	19	76	GCA_000167895.3
CB9615	Missing	Germany	-	1	5,386,352	50.5	5325	22	100	GCA_000025165.1
E110019	Missing	Missing	-	1	5,249,232	50.8	5311	22	92	GCA_000167875.1
90-9269	1988	Bangladesh	-	1	4,760,041	50.7	4731	22	89	GCA_002764155.1

90-9276	1988	Bangladesh	-	1	5,004,671	50.7	4983	22	91	GCA_002741235.1
90-9280	1988	Bangladesh	-	1	4,966,338	50.7	4880	22	91	GCA_002741255.1
90-9281	1988	Bangladesh	-	1	4,978,613	50.6	4881	22	89	GCA_002741455.1
214-4	1974	Mexico	-	1	5,138,709	50.7	5173	22	91	GCA_003956245.1
ATCC-43886	Missing	Missing	-	1	4,914,654	50.8	4896	22	96	GCA_002741295.1
B7A	Missing	Missing	-	289	5,300,242	50.7	5510	17	65	GCA_000167815.1
E24377A	Missing	Missing	-	1	4,979,619	50.6	4875	22	91	GCA_000017745.1
H10407	Missing	Missing	-	1	5,153,435	50.8	5147	22	90	GCA_000210475.1
UMNK88	Missing	USA	-	1	5,186,416	50.7	5255	22	96	GCA_000212715.2
ATCC-43889	Missing	USA	-	3	5,753,142	50.4	5981	22	108	GCA_006874785.1
E2865	2014	Japan	-	1	5,678,205	50.7	5914	22	100	GCA_003966465.1
E32511	Missing	Missing	-	1	5,471,440	50.5	5562	22	103	GCA_013167875.1
FORC_028	2003-01-01	South Korea	-	1	5,704,396	50.6	5919	22	94	GCA_001721125.1
RM8426	2009-01	USA	-	1	5,648,177	50.7	5857	22	101	GCA_003112185.1
RM13514	Missing	USA	-	1	5,585,613	50.7	5714	22	105	GCA_000520035.1

CDS, coding sequence
